# Microwave-Assisted Synthesis and Antimicrobial Evaluation of Novel Spiroisoquinoline and Spiropyrido[4,3-*d*]pyrimidine Derivatives

**DOI:** 10.3390/molecules20021842

**Published:** 2015-01-23

**Authors:** Rasha M. Faty, Mohamed S. Rashed, Mohamed M. Youssef

**Affiliations:** 1 Chemistry Department, Faculty of Science, Cairo University, Cairo 12613, Egypt; E-Mail: rashafaty@yahoo.com; 2 Schlumberger, Well Services, Al-Khobar 31952, Saudi Arabia; E-Mail: MHassan2@slb.com

**Keywords:** bromination, homophthalimide, pyrido[4,3-*d*]pyrimidine, spiro compounds, microwave, antimicrobial

## Abstract

Bromination of *N*-substituted homophthalimides and tetrahydropyrido[4,3-*d*]-pyrimidine-5,7-diones produces 4,4-dibromohomophthalimide and 8,8-dibromo-tetrahydropyrido[4,3-*d*]pyrimidine-5,7-dione derivatives, respectively, that can be used as precursors for spiro derivatives. The dibromo derivatives react with different binucleophilic reagents to produce several spiroisoquinoline and spirotetrahydropyrido[4,3-*d*]-pyrimidine-5,7-dione derivatives, respectively. Reaction of the dibromo derivatives with malononitrile produces dicyanomethylene derivatives which react with different binucleophiles to produce new spiro derivatives. All new compounds are prepared by using the usual chemical conditions and microwave assisted conditions. The latter conditions improved the reaction yields, reduced reaction times and ameliorated the effects on the surrounding environment as the reactions are carried out in closed systems. Structures of the newly synthesized compounds are proved using spectroscopic methods such as IR, MS, ^1^H-NMR and ^13^C-NMR and elemental analyses. Some of the newly synthesized compounds were tested for their antimicrobial activities, whereby four of them showed moderate activities and the rest showed low or no activities towards the investigated species.

## 1. Introduction

Spiro compounds constitute a group of generally less investigated compounds, however, recently growing efforts have been made to synthesize and characterize these compounds. Many spiro compounds possess very promising biological activities as anticancer [1,2], antibacterial [3,4], anticonvulsant [5,6,7], antituberculosis [8], anti-Alzheimer’s [9], pain-relief [10,11] and antidermatitis agents [12]. In addition to their medical uses, some spiro-compounds have found other uses in the agricultural and industrial fields. For example, they are used as antifungal agents [13], pesticides [14], laser dyes [15] and electroluminescent devices [16]. Spiro compounds have also been used as antioxidants [17,18]. Our research group is interested in using the microwave technique [3,19,20,21,22,23,24,25], as it has several advantages over conventional methods of synthesis, such as reduced reaction times, fewer effects on the environment and better reactions. In the present research, we used both the microwave technique as well as conventional methods to prepare some new spiro compounds that were then tested for their antimicrobial activities.

## 2. Results and Discussion

### 2.1. Chemistry

**Scheme 1:**
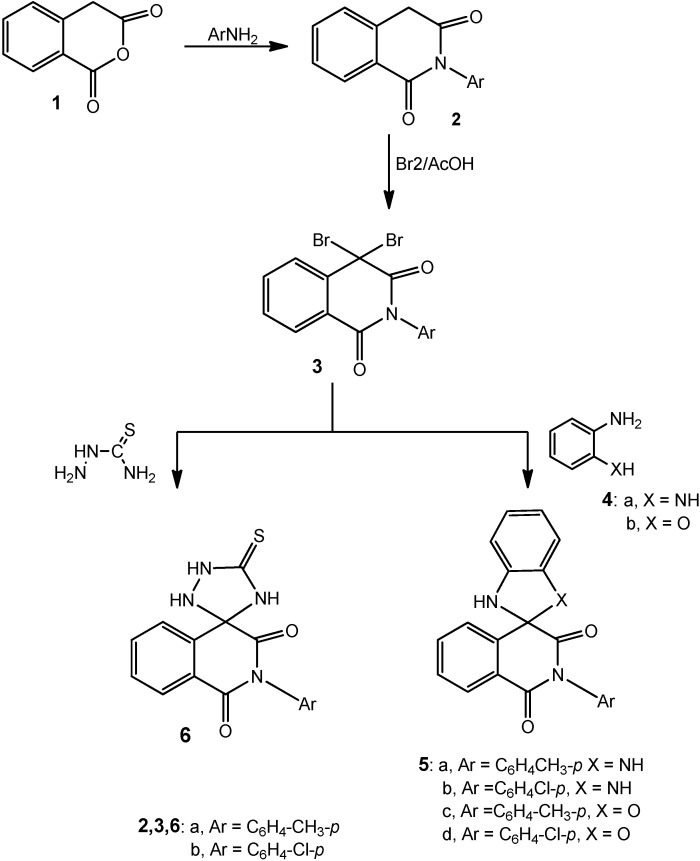
Reactions of dibromohomophthalimides with binucleophilic reagents: synthesis of **5** and **6**.

Homophthalic anhydride (**1**) was reacted with aromatic amines, namely *p*-toluidine and *p*-chloroaniline, to afford *N*-arylhomophthalimide derivatives **2a**,**b** respectively, which were used as precursors for preparing new spiroisoquinolines (Scheme 1). Compounds **2a**,**b**, having an active methylene group, reacted with two equivalents of bromine in acetic acid to produce 2-aryl-4,4-dibromoisoquinoline-1,3-(2*H*,4*H*)dione derivatives **3a**,**b**. The mass spectrum of compound **3a** displayed the expected molecular ion isomeric peaks at *m/z* 407 (4.8%), 409 (10.1%), 411 (5.2%). Compound **3b** gave the molecular ion peaks at 427 (4.4%), 429 (9.9%), 431 (6.9%).

Compounds **3a**,**b** underwent direct cyclocondensation when treated with each of *o*-phenylenediamine (**4a**) or *o*-aminophenol (**4b**) to produce 2'-aryl-1,3-dihydro-1'*H*-spiro-[benzo[d]imidazole-2,4'-isoquinoline]-1',3'(2'*H*)-diones **5a**,**b** and 2'-aryl-1'*H*,3*H*-spiro[benzo[d]oxazole-2,4'-isoquinoline]-1',3'(2'*H*)-diones **5c**,**d**, respectively (Scheme 1). The synthesis of compounds **5a**–**d** was carried out under conventional heating conditions. Thus, when the reaction was carried out in a refluxing ethanolic piperidine solution for 5 h under TLC monitoring, the product **5a**–**d** were obtained in 42%–51% yields.

Similarly, compounds **3a**,**b** reacted with thiosemicarbazide under the same reaction conditions and produced 2-aryl-5'-thioxo-1*H*-spiro[isoquinoline-4,3'-[1,2,4]triazolidine]-1,3(2*H*)-diones **6a**,**b** (Scheme 1). The analytical and spectral data of **5a**–**d** and **6a**,**b** were in agreement with the proposed structures (Experimental Section).

In a similar manner, when 4-(4-aryl)-6-phenyl-2-thioxo-2,3,4,4a-tetrahydropyrido-[4,3-*d*]-pyrimidine-5,7(6*H*,8*H*)-diones **7a**,**b** [26] were treated with two equivalents of bromine in acetic acid, the 8,8-dibromo derivatives **8a**,**b** were obtained. Elemental analyses as well as the spectroscopic data of **8a**,**b** agreed with the proposed structures (Experimental Section). Heating under reflux compounds **8a**,**b** in absolute ethanol in presence of piperidine with either of ethylenediamine or thiosemicarbazide afforded the corresponding targeted spiro compounds 4'-(4-aryl)-6'-phenyl-2'-thioxo-3',4'-dihydro-1'*H*-spiro- [imidazolidine-2,8'-pyrido[4,3-*d*]pyrimidine]-5',7'(2'*H*,6'*H*)-diones **9a**,**b** and 4-(4-aryl)-6-phenyl-2,5'- dithioxo-3,4-dihydro-1*H*-spiro[pyrido[4,3-*d*]pyrimidine-8,3'-[1,2,4]triazolidine]-5,7(2*H*,6*H*)-diones **10a**,**b**, respectively.

**Scheme 2:**
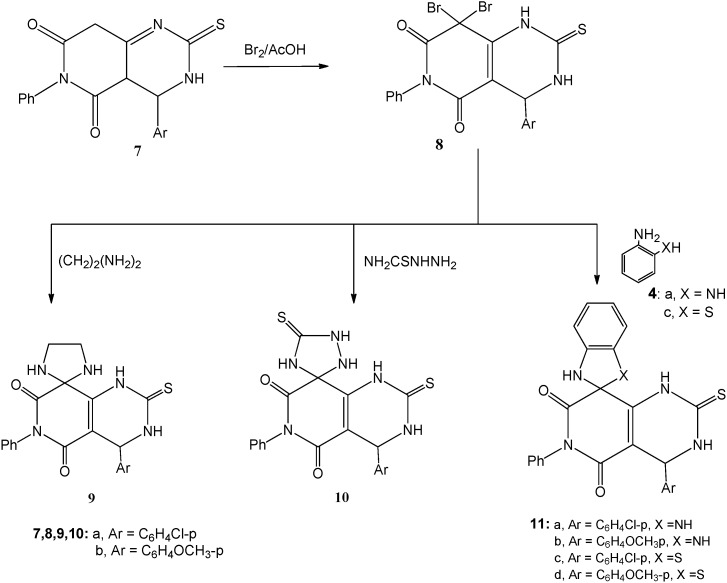
Reaction of dibromopyridopyrimidines **8a**,**b** with binucleophilic reagents; formation of **9**, **10** and **11**.

Similarly, compounds **8a**,**b** were refluxed with *o*-phenylenediamine (**4a**) and *o*-aminothiophenol (**4c**) in absolute ethanol, in the presence of piperidine to afford the corresponding cyclized products 4'-(4-aryl)-6'-phenyl-2'-thioxo-1,3,3',4'-tetrahydro-1'*H*-spiro[benzo[*d*]-imidazole-2,8'-pyrido[4,3-*d*]pyrimidine]-5',7'-(2'*H*,6'*H*)-diones **11a**,**b** and 4'-(4-aryl)-6'-phenyl-2'-thioxo-3',4'-dihydro-1'*H*,3*H*-spiro-[benzo[*d*]thiazole-2,8'-pyrido-[4,3-*d*]pyrimidine]-5',7'(2'*H*, 6'*H*)-diones **11c**,**d**, respectively (Scheme 2). The produced compounds **9a**,**b**, **10a**,**b** and **11a–d** gave fully consistent elemental and spectroscopic analyses data (Experimental Section).

On the other hand, Refluxing compounds **8a**,**b** in ethanol/piperidine with either with malononitrile (**12a**) or ethyl cyanoacetate (**12b**) afforded the corresponding 2-(4-(4-aryl)-5,7-dioxo-6-phenyl-2-thioxo-1,2,3,4,6,7-hexahydropyrido[4,3-*d*]pyrimidin-8(5*H*)-ylidene)malononitriles (compounds **13a**,**b**) and ethyl 2-(4-(4-aryl)-5,7-dioxo-6-phenyl-2-thioxo-1,2,3,4,6,7-hexahydropyrido-[4,3-*d*]pyrimidin- 8(5*H*)-ylidene)-2-cyanoacetates **13c**,**d**, respectively.

**Scheme 3:**
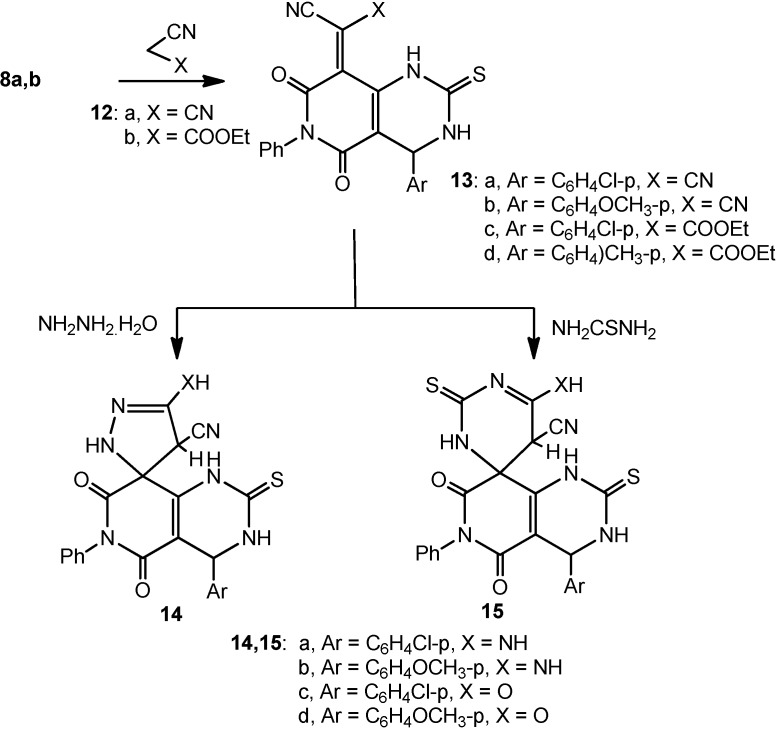
Reaction of **8a**,**b** with activated methylene compounds followed by binucleophiles; formation of **14a**–**d** and **15a**–**d**.

Compounds **13a**–**d** were refluxed with hydrazine hydrate in ethanol to obtain the 5-substituted-4'-(4-aryl)-5',7'-dioxo-6'-phenyl-2'-thioxo-2,2',3',4,4',5',6',7'-octahydro-1'*H*-spiro[pyrazole-3,8'-pyrido-[4,3-*d*]pyrimidine]-4-carbonitrile spiro products **14a**–**d**, respectively. Similar treatment of compounds **13a**–**d** with thiourea in ethanol/piperidine resulted in the formation of the 6'-substituted-4-(4-aryl)-5,7-dioxo-6-phenyl-2,2'-dithioxo-2,3,3',4,5,5',6,7-octahydro-1*H*,2'*H*-spiro-[pyrido[4,3-*d*]pyrimidine-8,4'-pyrimidine]-5'-carbonitriles **15a–d**, respectively (Scheme 3).

Trying to take advantage of the benefits of the microwave assisted reaction conditions, compounds **5**, **6**, **9**, **10**, **11**, **14** and **15** were prepared by using microwave irradiation instead of the conventional heating conditions. The results showed that much less time was needed to prepare these compounds, as well as a considerable increase in the reaction yields upon using the environmentally friendly microwave irradiation conditions. Table 1 shows a comparison in reaction times and yields between the conventional and microwave assisted methods of preparation.

**Table 1:** Comparison between conventional methods and microwave assisted methods of synthesis of compounds **5a**–**d**, **6a**,**b**, **9a**,**b**, **10a**,**b**, **11a**–**d**, **14a**–**d** and **15a**–**d**.

Compound No.	Reaction Times		Reaction Yields (%)	
	Conventional Methods *	Microwave ^‡^	Conventional Methods *	Microwave ^‡^
**5a**	5 h	15 min	42	91
**5b**	5 h	15 min	48	89
**5c**	5 h	15 min	47	82
**5d**	5 h	15 min	53	92
**6a**	4 h	10 min	60	94
**6b**	4 h	10 min	55	90
**9a**	2 h	5 min	65	93
**9b**	2 h	5 min	67	91
**10a**	2 h	5 min	61	91
**10b**	2 h	5 min	58	89
**11a**	2 h	5 min	47	84
**11b**	2 h	5 min	50	91
**11c**	2 h	5 min	52	88
**11d**	2 h	5 min	54	80
**14a**	3 h	7 min	37	81
**14b**	3 h	7 min	42	84
**14c**	3 h	7 min	45	89
**14d**	3 h	7 min	44	82
**15a**	3 h	7 min	47	88
**15b**	3 h	7 min	43	87
**15c**	3 h	7 min	51	90
**15d**	3 h	7 min	47	91

* Conventional reaction conditions: the reactants were heated under reflux in the proper solvent for 2–5 h in open systems (Experimental Section). **^‡^** Microwave-assisted reaction conditions: the reactants were heated in tightly closed tubes in scientific microwave oven for 5–15 min (Experimental Section).

### 2.2. Antimicrobial Evaluation

The newly synthesized heterocyclic compounds were tested for their antimicrobial activity against the following microorganisms: *Escherichia coli*, *Pseudomonas putida*, *Bacillus subtilis*, *Streptococcus lactis*, *Aspergillus niger*, *Penicillium* sp. and *Candida albicans*. The filter paper disc diffusion method [27] was used to perform preliminary screening of the investigated compounds. The most active compounds were **5a**, **6b**, **9a** and **9b**, which showed moderate inhibition to the microorganisms. Also, compounds **5c**, **6a**, **11a**, **11c** and **15b** showed slight inhibitory action. The rest of compounds showed no sensitivity at all to the tested organisms, and the results are summarized in Table 2.

**Table 2:** Antimicrobial activities of the newly synthesized compounds.

Comp. No.	Inhibition Zone (mm)						
	Gram-Negative		Gram-Positive		Fungi		Yeast
	*E. coli*	*P. putida*	*B. subtilis*	*S. lactis*	*A. niger*	*P.* sp.	*C. albicans*
**5a**	11	13	8	10	9	8	0
**5c**	4	4	3	2	4	0	0
**6a**	8	6	7	8	6	6	0
**6b**	14	15	13	13	11	9	0
**9a**	10	10	11	10	10	8	0
**9b**	12	10	10	9	6	5	0
**10a**	0	0	0	0	0	0	0
**10b**	0	0	0	0	0	0	0
**11a**	5	5	3	3	5	4	0
**11c**	7	8	7	6	4	0	0
**14a**	0	0	0	0	0	0	0
**14d**	0	0	0	0	0	0	0
**15b**	7	5	5	4	6	3	0
**15c**	0	0	0	0	0	0	0
Chloram-phenicol	22	21	18	19	20	12	0
Ampicillin	24	20	19	22	24	14	14

*E. coli* = *Escherichia coli*; *P. putida* = *Pseudomonas putida*; *B. subtilis* = *Bacillus subtilis*; *S. lactis* = *Streptococcus lactis*; *A. niger* = *Aspergillus niger*; *P.* sp. = *Penicillium* sp.; *C. albicans* = *Candida albicans*. The sensitivity of microorganisms to the tested compounds is identified in the following manner: Highly sensitive = Inhibition zone 15–20 mm; Moderately sensitive = Inhibition zone: 10–15 mm; Slightly sensitive = Inhibition zone: 5–10 mm; Not sensitive = Inhibition zone: 0 mm; Each result represents the average of triplicate readings.

## 3. Experimental Section

### 3.1. General

Melting points were determined in open glass capillaries on a Gallenkamp melting point apparatus and are uncorrected. IR spectra (KBr discs) were recorded on Shimadzu FTIR-8201PC spectrophotometer (Giza, Egypt). ^1^H-NMR and ^13^C-NMR spectra were recorded on a Varian Mercury 300 MHz or Varian Gemini 200 MHz spectrometers (Giza, Egypt) using TMS as an internal standard and DMSO-*d*_6_ as solvent. Microwave reactions were performed with a Millstone Organic Synthesis Unit with touch control terminal (MicroSYNTH, Giza, Egypt) with a continuous focused microwave power delivery system in a pressure glass vessel (10 mL) sealed with a septum under magnetic stirring. The temperature of the reaction mixture was monitored using a calibrated infrared temperature control under the reaction vessel, and control of the pressure was performed with a pressure sensor connected to the septum of the vessel. Elemental analysis was carried out at the Microanalytical Center of Cairo University, Giza, Egypt.

#### 3.1.1. 2-Aryl-4,4-dibromoisoquinoline-1,3-(2*H*,4*H*)dione Derivatives **3a**,**b**

A solution of either of **2a** (2.37 g, 0.01 mol), **2b** (2.51 g, 0.01 mol) or **2c** (2.72 g, 0.01 mol) in glacial acetic acid (20 mL) was heated under reflux with bromine (1.1 mL, 3.0 g, 0.02 mole) for 2 h. After cooling, the reaction mixture was poured onto ice-water and the solid that precipitated was filtered off, dried and crystallized from the proper solvent.

*4,4-Dibromo-2-p-tolylisoquinoline-1,3-(2H,4H)dione* (**3a**): white crystals after crystallization from acetic acid then washing with ethanol; 66% yield; m.p. 236–238 °C; ^1^H-NMR: 2.60 (s, 3H, CH_3_), 7.10–8.30 (m, 8H, Ar-H); ^13^C-NMR: 25.3 (CH_3_), 80.2 (sp3 C-4), 118.2, 122.7, 125.3, 126.1, 128.7, 130.0, 131.5, 134.6, 135.2, 136.4 (aromatic C), 158.4, 167.6 (2 CO); IR ν: 3066 cm^−1^ (aromatic CH), 2970 (aliphatic CH), 1645 (broad, 2C=O), 1605, 1500 (aromatic C=C); MS: M^+^ *m/z* 407 (3.2%), 409 (6.7%), 411 (3.0%); Anal. Calcd. for C_16_H_11_Br_2_NO_2_ (407.07): C (46.98%), H (2.71%), Br (39.07%), N (3.42%); Found: C (46.7%), H (2.9%), Br (38.93%), N (3.1%).

*4,4-Dibromo-2-(4-chlorophenyl)isoquinoline-1,3(2H,4H)-dione* (**3b**): white crystals after crystallization from acetic acid then washing with ethanol; 52% yield; m.p. 216–218 °C; ^1^H-NMR: 7.40–8.50 (m, 8H, Ar-H); ^13^C-NMR: 80.2 (sp3 C-4), 121.5, 123.9, 125.8, 127.1, 128.7, 130.0, 131.5, 134.6, 135.2, 136.4 (aromatic C), 158.2, 167.5 (2 CO); IR ν: 3060 cm^−1^ (aromatic CH), 1645 (broad, 2C=O), 1605, 1500 (aromatic C=C); MS: M^+^ *m/z* 427 (4.7%), 429 (10.3%), 431 (7.2%); Anal. Calcd.forC_15_H_8_Br_2_ClNO_2_ (429.49): C (41.95%), H (1.88%), Br (37.21%), Cl (8.25%), N (3.26%); Found: C (41.7%), H (1.7%), Br (37.1%), Cl (8.4%), N (3.1%).

#### 3.1.2. Cyclocondensation of **3a** with *o*-Phenylenediamine and *o*-Aminophenol; Formation of **5a**–**d**

**Method A**: Compounds **3a**,**b** (0.01 mol) were heated under reflux with either of *o*-phenylenediamine (1.08 g, 0.01 mol) or *o*-aminophenol (1.09 g, 0.01 mol) in absolute ethanol (25 mL) and few drops of piperidine for 5 h. The reaction mixture was then cooled, acidified with few drops of conc. hydrochloric acid and the solid that precipitated was filtered at the pump and crystallized from the appropriate solvent.

**Method B**: The same reactants of method A were heated in microwave oven at 500 W and 140 °C for 15 min. The reaction mixture was treated similar to method A to obtain compounds **5a**–**d**.

*2'-(4-Tolyl)-1,3-dihydro-1'H-spiro[benzimidazole-2,4'-isoquinoline]-1',3'(2'H)-dione* (**5a**): grey crystals after crystallization from acetic acid; 42% yield (Method A) and 91% (Method B); 229–231 °C; ^1^H-NMR: 2.40 (s, 3H, CH_3_), 3.80 (s, 2H, 2NH, D_2_O exchangeable), 6.40–7.70 (m, 12H, Ar-H); ^13^C-NMR: 25.5 (CH_3_), 83.4 (sp3-spiro C), 113.0, 115.9, 120.2, 122.9, 127.3, 127.8, 128.3, 128.9, 131.2, 133.7, 135.1, 135.9, 136.8 (aromatic C), 155.4, 163.6 (2 CO); IR ν: 3180 cm^−1^ (broad, NH), 3065 (aromatic CH), 2970 (aliphatic CH), 1655, 1640 (2C=O), 1605, 1500 (aromatic C=C); MS: M^+^ *m/z* 355 (12.3%); Anal. Calcd. for C_22_H_17_N_3_O_2_ (355.39): C (74.35%), H (4.82%), N (11.82%); Found: C (74.1%), H (4.4%), N (12.1%).

*2'-(4-Chlorophenyl)-1,3-dihydro-1'H-spiro[benzimidazole-2,4'-isoquinoline]-1',3'(2'H)-dione* (**5b**): grey crystals after crystallization from acetic acid; 48% yield (Method A) and 89% (Method B); m.p. 215–217 °C; ^1^H-NMR: 3.90 (s, 2H, 2NH, D_2_O exchangeable), 6.90–8.10 (m, 12H, Ar-H); ^13^C-NMR: 83.4 (sp3-spiro C), 115.0, 118.1, 123.6, 125.2, 127.3, 128.8, 129.6, 130.9, 131.8, 133.7, 135.1, 135.9, 136.8 (aromatic C), 155.4, 163.6 (2 CO); IR ν: 3180 cm^−1^ (broad, NH), 3065 (aromatic CH), 1655, 1640 (2C=O), 1605, 1500 (aromatic C=C); MS: M^+^ *m/z* 375 (11.5%), 377 (4.1%); Anal. Calcd. for C_21_H_14_ClN_3_O_2_ (375.81): C (67.12%), H (3.75%), Cl (9.43%), N (11.18%); Found: C (76.0%), H (3.5%), Cl (9.3%), N (11.3%).

*2'-p-Tolyl-1,3-dihydro-1'H-spiro[benzo[d]imidazole-2,4'-isoquinoline]-1',3'(2'H)-dione* (**5c**): grey crystals after crystallization from acetic acid; 47% yield (Method A) and 82% (Method B); m.p. 240–242 °C; ^1^H-NMR: 2.60 (s, 3H, CH_3_), 3.90 (s, 1H, NH, D_2_O exchangeable), 6.70–7.90 (m, 12H, Ar-H); ^13^C-NMR: 83.4 (sp3-spiro C), 115.4, 118.8, 122.7, 126.2, 127.3, 128.8, 129.6, 130.9, 131.8, 133.2, 136.8, 137.9, 142.8 (aromatic C), 155.4, 163.6 (2 CO); IR ν: 3190 cm^−1^ (broad, NH), 3065 (aromatic CH), 2975 (aliphatic CH), 1655,1645 (2C=O), 1605, 1500 (aromatic C=C); MS: M^+^ *m/z* 356 (10.3%); Anal. Calcd. for C_22_H_16_N_2_O_3_ (356.37): C (74.15%), H (4.53%), N (7.86%); Found: C (74.05%), H (4.43%), N (7.75%).

*2'-(4-Chlorophenyl)-1'H,3H-spiro[benzo[d]oxazole-2,4'-isoquinoline]-1',3'(2'H)-dione* (**5d**): grey crystals after crystallization from acetic acid; 53% yield (Method A) and 92% (Method B); m.p. 220–222 °C; ^1^H-NMR: 3.90 (s, 1H, NH, D_2_O exchangeable), 6.70–8.00 (m, 12H, Ar-H); ^13^C-NMR: 93.8 (sp3-spiro C), 119.0, 122.1, 124.9, 125.8, 127.5, 128.9, 129.6, 131.2, 132.9, 134.7, 137.1, 139.9, 146.8 (aromatic C), 155.4, 163.6 (2 CO); IR ν: 3150 cm^−1^ (broad, NH), 3065 (aromatic CH), 1655,1645 (2C=O), 1605, 1500 (aromatic C=C); MS: M^+^ *m/z* 376 (8.5%), 378 (2.7%); Anal. Calcd. for C_21_H_13_ClN_2_O_3_ (376.79): C (66.94%), H (3.48%), Cl (9.41%), N (7.43%); Found: C (66.6%), H (3.6%), Cl (9.1%), N (7.3%).

#### 3.1.3. Cyclocondensation of **3a**,**b** with Thiosemicarbazide; Formation of **6a**,**b**

**Method A**: Each of *c*ompounds **3a**,**b** (0.01 mol), was heated under reflux with thiosemicarbazide (0.91 g, 0.01 mol), absolute ethanol (25 mL) and few drops of piperidine for 4 h. The reaction mixture was then cooled, acidified with few drops of conc. hydrochloric acid and the solid that precipitated was filtered at the pump and crystallized from the appropriate solvent.

**Method B**: The same reactants of method A were heated in microwave oven at 500 W and 140 °C for 10 min. The reaction mixture was treated similar to method A to obtain compounds **6a**,**b**.

*5'-Thioxo-2-p-tolyl-1H-spiro[isoquinoline-4,3'-[1,2,4]triazolidine]-1,3(2H)-dione* (**6a**): white crystals after crystallization from acetic acid and washing with ethanol; 55% yield (Method A) and 90% (Method B); m.p. 156–158 °C; ^1^H-NMR: 2.40 (s, 3H, CH_3_), 3.10 (s, 1H, NH, D_2_O exchangeable), 3.60 (s, 1H, NH, D_2_O exchangeable), 4.00 (s, 1H, NH, D_2_O exchangeable), 7.20–7.90 (m, 8H, Ar-H); ^13^C-NMR: 23.2 (CH_3_), 91.7 (sp3 spiro C), 121.1, 123.4, 127.6, 128.4, 128.8, 129.8, 132.1, 134.0, 135.2, 136.8 (aromatic C), 154.9, 159.5 (2 CO), 176.9 (CS); IR ν: 3220, 3185, 3150 cm^−1^ (NH), 3060 (aromatic CH), 2970 (aliphatic CH), 1670,1650 (2C=O), 1600, 1490 (aromatic C=C); MS (70 eV): M^+^ *m/z* 338 (10.3%); Anal. Calcd. for C_17_H_14_N_4_O_2_S (338.36): C (60.34%), H (4.17%), N (16.56%), S(9.48%); Found: C (60.0%), H (3.9%), N (16.8%), S (9.7%).

*2-(4-Chlorophenyl)-5'-thioxo-1H-spiro[isoquinoline-4,3'-[1,2,4]triazolidine]-1,3(2H)-dione* (**6b**): white crystals after crystallization from dilute acetic acid and washing with ethanol; 60% yield (Method A) and 94% (Method B); m.p. 188–190 °C; ^1^H-NMR: 3.10 (s, 1H, NH, D_2_O exchangeable), 3.50 (s, 1H, NH, D_2_O exchangeable), 4.00 (s, 1H, NH, D_2_O exchangeable), 7.20–7.90 (m, 8H, Ar-H); ^13^C-NMR: 92.7 (sp3 spiro C), 127.1, 127.9, 132.6, 133.4, 134.8, 135.6, 136.1, 137.0, 137.9, 138.8 (aromatic C), 156.9, 160.7 (2CO), 180.1 (CS); IR ν: 3220, 3185, 3150 cm^−1^ (NH), 3060 (aromatic CH), 1670,1645 (2C=O), 1600, 1490 (aromatic C=C); MS (70 eV): M^+^ *m/z* 358 (12.7%) and 360 (4.3%); Anal. Calcd. for C_16_H_11_N_4_O_2_S (358.80): C (53.56%), H (3.09%), Cl (9.88%), N (15. 61%), S (8.94%); Found: C (53.4%), H (2.90%), Cl (9.6%), N (15.5%), S (8.7%).

#### 3.1.4. Bromination of **7a**,**b**; Formation of **8a**,**b**

A solution of either of **7a** or **7b** (0.01 mol) in ethanol (20 mL) was heated under reflux with bromine (1.1 mL, 3.0 g, 0.02 mol) for 2 h. After cooling, the reaction mixture was poured onto ice-water and the solid that precipitated was filtered off, dried and crystallized from the proper solvent.

*8,8-Dibromo-4-(4-chlorophenyl)-6-phenyl-2-thioxo-1,2,3,4-tetrahydropyrido[4,3-d]pyrimidine-5,7(6H,8H)-dione* (**8a**): white crystals after crystallization from absolute ethanol; 55% yield; m.p. 255–257 °C; ^1^H-NMR: 3.00 (s, 1H, NH, D_2_O exchangeable), 5.40 (s, 1H, CH), 7.20–7.70 (m, 9H, Ar-H), 11.30 (s, 1H, NH, D_2_O exchangeable); ^13^C-NMR: 55.7 (pyrimidine C-4), 80.9 (pyridine C-8), 127.1, 127.9, 132.6, 133.4, 134.8, 135.6, 136.1, 137.0, 139.9, 150.8 (aromatic C), 156.9, 160.7 (2CO), 178.0 (CS); IR ν: 3220, 3180 cm^−1^ (NH), 3060 (aromatic CH), 2850 (aliphatic CH), 1670, 1640 (2C=O), 1600, 1490 (aromatic C=C); MS (70 eV): M^+^ *m/z* 539 (4.7%), 541 (10.7%) and 543 (7.1%); Anal. Calcd. for C_19_H_12_Br_2_ClN_3_O_2_S (541.64): C (42.13%), H (2.23%), Br (29.50%), Cl (6.55%), N (7.76%), S (5.92%); Found: C (42.0%), H (2.3%), Br (29.4%), Cl (6.7%), N (7.9%), S (5.8%).

*8,8-Dibromo-4-(4-methoxyphenyl)-6-phenyl-2-thioxo-1,2,3,4-tetrahydropyrido[4,3-d]pyrimidine-5,7(6H,8H)-dione* (**8b**): white crystals after crystallization from absolute ethanol; 53% yield; m.p. 232–234 °C; ^1^H-NMR: 3.00 (s, 1H, NH, D_2_O exchangeable), 3.80 (s, 3H, OCH_3_), 5.40 (s, 1H, CH), 6.90–7.60 (m, 9H, Ar-H), 11.30 (s, 1H, NH, D_2_O exchangeable); ^13^C-NMR: 53.9 (OCH_3_), 55.5 (pyrimidine C-4), 83.1 (pyridine C-8), 127.4, 128.4, 132.7, 133.8, 134.9, 136.6, 137.1, 138.5, 148.3, 150.8 (aromatic C), 156.9, 160.7 (2CO), 182.0 (CS); IR ν: 3220, 3180 cm^−1^ (NH), 3060 (aromatic CH), 2880 (aliphatic CH), 1665, 1645 (2C=O), 1600, 1490 (aromatic C=C); MS (70 eV): M^+^ *m/z* 535 (6.5%), 537 (13.7%) and 539 (6.1%); Anal. Calcd. for C_20_H_15_Br_2_N_3_O_3_S (537.22): C (44.71%), H (2.81%), Br (29.75%), N (7.82%), S (5.97%); Found: C (44.8%), H (2.8%), Br (29.9%), N (7.7%), S (6.1%).

#### 3.1.5. Cyclocondensation of **8a**,**b** with Ethylene Diamine and Thiosemicarbazide; Formation of **9a** and **10a**

**Method A**: Each of compounds **8a**,**b** (0.01 mol) was heated under reflux with either of ethylene diamine (0.67 mL, 0.01 mol) or thiosemicarbazide (0.91 g, 0.01 mol) in absolute ethanol (25 mL)] and few drops of piperidine for 2 h. The reaction mixture was then cooled, acidified with few drops of conc. hydrochloric acid and the solid that precipitated was filtered at the pump and crystallized from the appropriate solvent to give **9a**,**b** and **10a**,**b**.

**Method B**: The same reactants of method A were heated in microwave oven at 500 W and 140 °C for 5 min. The reaction mixture was treated similar to method A to obtain compounds **9a**,**b** and **10a**,**b**.

*4'-(4-Chlorophenyl)-6'-phenyl-2'-thioxo-3',4'-dihydro-1'H-spiro[imidazolidine-2,8'-pyrido[4,3-d]pyrimidine]-5',7'(2'H,6'H)-dione* (**9a**): white crystals after crystallization from absolute ethanol; 65% yield (Method A) and 93% (Method B); m.p. 247–249 °C; ^1^H-NMR: 2.60 (s, 4H, 2CH_2_-imidazolidine), 3.10 (s, 1H, NH, D_2_O exchangeable), 3.80 (s, 2H, 2NH, D_2_O exchangeable), 5.30 (s, 1H, CH), 7.20–7.70 (m, 9H, Ar-H), 11.30 (s, 1H, NH, D_2_O exchangeable); ^13^C-NMR: 51.2 (imidazolidine 2CH_2_), 55.7 (pyrimidine C-4), 80.9 (pyridine C-8), 127.1, 127.9, 132.6, 133.4, 134.8, 135.6, 136.1, 137.0, 138.5, 147.3 (aromatic C), 156.9, 160.4 (2CO), 181.0 (CS); IR ν: 3280, 3220, 3180 cm^−1^ (NH), 3060 (aromatic CH), 2850 (aliphatic CH), 1670, 1645 (2C=O), 1600, 1490 (aromatic C=C); MS (70 eV): M^+^ *m/z* 439 (8.7%) and 441 (3.2%). Anal. Calcd. for C_21_H_18_ClN_5_O_2_S (439.92): C (57.33%), H (4.12%), Cl (8.06%), N (15.92%), S (7.29%); Found: C (57.1%), H (4. 2%), Cl (7.9%), N (16.1%), S (7.1%).

*4'-(4-Methoxyphenyl)-6'-phenyl-2'-thioxo-3',4'-dihydro-1'H-spiro[imidazolidine-2,8'-pyrido[4,3-d]pyrimidine]-5',7'(2'H,6'H)-dione* (**9b**): white crystals after crystallization from absolute ethanol; 67% yield (Method A) and 91% (Method B); m.p. 215–217 °C; ^1^H-NMR: 2.60 (s, 4H, 2CH_2_-imidazolidine), 3.00 (s, 1H, NH, D_2_O exchangeable), 3.60 (s, 2H, 2NH, D_2_O exchangeable), 3.80 (s, 3H, OCH_3_), 5.40 (s, 1H, CH), 6.90–7.60 (m, 9H, Ar-H), 11.30 (s, 1H, NH, D_2_O exchangeable); ^13^C-NMR: 51.0 (imidazolidine 2CH_2_), 53.9 (OCH_3_), 55.5 (pyrimidine C-4), 83.1 (pyridine C-8), 116.4, 118.5, 125.7, 128.0, 134.9, 136.6, 137.1, 138.5, 148.3, 150.8 (aromatic C), 156.9, 160.7 (2CO), 182.0 (CS); IR ν: 3280, 3220, 3180 cm^−1^ (NH), 3060 (aromatic CH), 2880 (aliphatic CH), 1670, 1645 (2C=O), 1600, 1490 (aromatic C=C); MS (70 eV): M^+^ *m/z* 435 (7.1%); Anal. Calcd. for C_22_H_21_N_5_O_3_S (435.50): C (60.67%), H (4.86%), N (16.08%), S (7.36%); Found: C (60.7%), H (4.9%), N (15.9%), S (7.1%).

*4-(4-Chlorophenyl)-6-phenyl-2,5'-dithioxo-3,4-dihydro-1H-spiro[pyrido[4,3-d]pyrimidine-8,3'-[1,2,4]triazolidine]-5,7(2H,6H)-dione* (**10a**): white crystals after crystallization from absolute ethanol; 61% yield (Method A) and 91% (Method B); m.p. 225–227 °C; ^1^H-NMR: 3.00 (s, 1H, NH, D_2_O exchangeable), 3.10 (s, 1H, NH, D_2_O exchangeable), 3.40 (s, 1H, NH, D_2_O exchangeable), 5.30 (s, 1H, CH), 7.20–7.70 (m, 9H, Ar-H), 8.10 (s, 1H, NH, D_2_O exchangeable), 11.00 (s, 1H, NH, D_2_O exchangeable); ^13^C-NMR: 55.7 (pyrimidine C-4), 80.9 (pyridine C-8), 127.1, 127.9, 132.6, 133.4, 134.8, 135.6, 136.1, 137.0, 140.5, 152.3 (aromatic C), 156.9, 160.4 (2CO), 177.3, 181.0 (2CS); IR ν: 3280, 3220, 3180 cm^−1^ (NH), 3060 (aromatic CH), 2850 (aliphatic CH), 1675, 1640 (2C=O), 1600, 1490 (aromatic C=C); MS (70 eV): M^+^ *m/z* 470 (11.3%) and 441 (3.9%). Anal. Calcd. for C_20_H_15_ClN_6_O_2_S_2_ (470.96): C (51.01%), H (3.21%), Cl (7.53%), N (13.62%), S (7.29%); Found: C (50.9%), H (3.1%), Cl (7.3%), N (13.4%), S (7.4%).

*4-(4-Methoxyphenyl)-6-phenyl-2,5'-dithioxo-3,4-dihydro-1H-spiro[pyrido[4,3-d]pyrimidine-8,3'-[1,2,4]triazolidine]-5,7(2H,6H)-dione* (**10b**): white crystals after crystallization from absolute ethanol; 58% yield (Method A) and 89% (Method B); m.p. 206–208 °C; ^1^H-NMR: 3.00 (s, 1H, NH, D_2_O exchangeable), 3.10 (s, 1H, NH, D_2_O exchangeable), 3.60 (s, 1H, NH, D_2_O exchangeable), 3.80 (s, 3H, OCH_3_), 5.40 (s, 1H, CH), 6.90–7.60 (m, 9H, Ar-H), 8.00 (s, 1H, NH, D_2_O exchangeable), 11.30 (s, 1H, NH, D_2_O exchangeable); ^13^C-NMR: 53.3 (OCH_3_), 55.7 (pyrimidine C-4), 80.9 (pyridine C-8), 114.3, 115.9, 122.6, 126.4, 131.8, 135.6, 136.1, 137.0, 142.5, 150.3 (aromatic C), 160.9, 164.0 (2CO), 173.4, 181.0 (2CS); IR ν: 3280, 3220, 3180 cm^−1^ (NH), 3060 (aromatic CH), 2850 (aliphatic CH), 1675, 1640 (2C=O), 1600, 1490 (aromatic C=C); MS (70 eV): M^+^ *m/z* 466 (8.3%). Anal. calcd. for C_21_H_18_N_6_O_3_S_2_ (466.54): C (54.06%), H (3.89%), N (18.01%), S (13.75%); Found: C (53.9%), H (3.7%), N (17.9%), S (13.6%).

#### 3.1.6. Cyclocondensation of **8a**,**b** with *o*-Phenylenediamine (**4a**) and *o*-Aminothiophenol (**4c**); Formation of **11a**–**d**

**Method A**: Each of compounds **8a**,**b** (0.01 mol) was heated under reflux with either of *o*-phenylenediamine (**4a**; 1.08 g, 0.01 mol) or *o*-aminothiophenol (**4c**; 1.25 mL, 0.01 mol) in absolute ethanol (25 mL) and few drops of piperidine for 2 h. The reaction mixture was then cooled, acidified with few drops of conc. hydrochloric acid and the solid that precipitated was filtered at the pump and crystallized from the appropriate solvent.

**Method B**: The same reactants of method A were heated in microwave oven at 500 W and 140 °C for 5 min. The reaction mixture was treated similar to method A to obtain compounds **11a**–**d**.

*4'-(4-Chlorophenyl)-6'-phenyl-2'-thioxo-1,3,3',4'-tetrahydro-1'H-spiro[benzo[d]imidazole-2,8'-pyrido[4,3-d]pyrimidine]-5',7'(2'H,6'H)-dione* (**11a**): white crystals after crystallization from absolute ethanol; 47% yield (Method A) and 84% (Method B); m.p. 258–260 °C; ^1^H-NMR: 3.10 (s, 1H, NH, D_2_O exchangeable), 4.40 (s, 2H, 2NH, D_2_O exchangeable), 5.30 (s, 1H, CH), 6.60–7.50 (m, 13H, Ar-H), 12.10 (s, 1H, NH, D_2_O exchangeable); ^13^C-NMR: 55.7 (pyrimidine C-4), 80.9 (pyridine C-8), 116.8, 119.2, 127.1, 127.9, 132.6, 133.3, 134.6, 135.6, 136.1, 137.0, 138.5, 145.3, 151.4 (aromatic C), 160.9, 164.4 (2CO), 175.1 (CS); IR ν: 3280, 3220, 3180 cm^−1^ (NH), 3040 (aromatic CH), 2830 (aliphatic CH), 1665, 1645 (2C=O), 1600, 1490 (aromatic C=C); MS (70 eV): M^+^ *m/z* 487 (6.3%) and 489 (2.2%). Anal. Calcd. for C_25_H_18_ClN_5_O_2_S (487.96): C (61.54%), H (3.72%), Cl (7.27%), N (14.35%), S (6.57%); Found: C (61.3%), H (3.6%), Cl (7.1%), N (14.3%), S (6.4%).

*4'-(4-Methoxyphenyl)-6'-phenyl-2'-thioxo-1,3,3',4'-tetrahydro-1'H-spiro[benzo[d]imidazole-2,8'-pyrido[4,3-d]pyrimidine]-5',7'(2'H,6'H)-dione* (**11b**): white crystals after crystallization from absolute ethanol; 50% yield (Method A) and 91% (Method B); m.p. 226–228 °C; ^1^H-NMR: 3.10 (s, 1H, NH, D_2_O exchangeable), 3.80 (s, 3H, OCH_3_), 4.10 (s, 2H, 2NH, D_2_O exchangeable), 5.40 (s, 1H, CH), 6.60–7.50 (m, 13H, Ar-H), 12.20 (s, 1H, NH, D_2_O exchangeable); ^13^C-NMR: 55.3 (OCH_3_), 55.7 (pyrimidine C-4), 80.9 (pyridine C-8), 112.8, 116.2, 123.1, 124.9, 132.6, 133.3, 134.6, 135.6, 136.1, 137.0, 138.5, 145.4, 150.6 (aromatic C), 160.9, 164.4 (2CO), 175.1 (CS); IR ν: 3280, 3220, 3180 cm^−1^ (NH), 3040 (aromatic CH), 2830 (aliphatic CH), 1665, 1645 (2C=O), 1600, 1490 (aromatic C=C); MS (70 eV): M^+^ *m/z* 483 (6.8%). Anal. Calcd. for C_26_H_21_N_5_O_3_S (483.54): C (64.58%), H (4.38%), N (14.48%), S (6.63%); Found: C (64.5%), H (4.1%), N (14.6%), S (6.5%).

*4'-(4-Chlorophenyl)-6'-phenyl-2'-thioxo-3',4'-dihydro-1'H,3H-spiro[benzo[d]thiazole-2,8'-pyrido[4,3-d]pyrimidine]-5',7'(2'H,6'H)-dione* (**11c**): white crystals after crystallization from absolute ethanol; m.p. 230–232 °C, in 52% yield (Method A) and 88% (Method B); ^1^H-NMR: 3.10 (s, 1H, NH, D_2_O exchangeable), 4.50 (s, 1H, NH, D_2_O exchangeable), 5.30 (s, 1H, CH), 6.60–7.50 (m, 13H, Ar-H), 11.80 (s, 1H, NH, D_2_O exchangeable); ^13^C-NMR: 55.7 (pyrimidine C-4), 82.0 (pyridine C-8), 116.0, 119.6, 121.3, 125.2, 127.4, 128.5, 132.6, 133.3, 134.6, 135.6, 136.1, 137.0, 138.5, 140.3 145.3, 151.4 (aromatic C), 160.9, 164.4 (2CO), 175.1 (CS); IR ν: 3280, 3220, 3180 cm^−1^ (NH), 3040 (aromatic CH), 2830 (aliphatic CH), 1665, 1645 (2C=O), 1600, 1490 (aromatic C=C); MS (70 eV): M^+^ *m/z* 504 (9.8%) and 489 (3.7%). Anal. Calcd. for C_25_H_17_ClN_4_O_2_S_2_ (505.01): C (59.46%), H (3.39%), Cl (7.02%), N (11.09%), S (12.70%); Found: C (59.5%), H (3.2%), Cl (6.9%), N (11.2%), S (12.6%).

*4'-(4-Methoxyphenyl)-6'-phenyl-2'-thioxo-3',4'-dihydro-1'H,3H-spiro[benzo[d]thiazole-2,8'-pyrido[4,3-d]pyrimidine]-5',7'(2'H,6'H)-dione* (**11d**): white crystals after crystallization from absolute ethanol; 45% yield (Method A) and 80% (Method B); m.p. 210–212 °C; ^1^H-NMR: 3.10 (s, 1H, NH, D_2_O exchangeable), 3.80 (s, 3H, OCH_3_), 4.00 (s, 1H, NH, D_2_O exchangeable), 5.40 (s, 1H, CH), 6.60–7.50 (m, 13H, Ar-H), 12.20 (s, 1H, NH, D_2_O exchangeable); ^13^C-NMR: 55.3 (OCH_3_), 55.7 (pyrimidine C-4), 80.9 (pyridine C-8), 116.4, 118.2, 121.2, 125.6, 127.1, 128.5, 132.6, 133.3, 134.6, 135.6, 136.1, 137.0, 138.5, 140.3, 145.1, 151.3 (aromatic C), 160.9, 164.4 (2CO), 175.1 (CS); IR ν: 3280, 3220, 3180 cm^−1^ (NH), 3040 (aromatic CH), 2830 (aliphatic CH), 1665, 1645 (2C=O), 1600, 1490 (aromatic C=C); MS (70 eV): M^+^ *m/z* 500 (11.3%). Anal. Calcd. for C_26_H_20_N_4_O_3_S_2_ (500.59): C (62.38%), H (4.03%), N (11.19%), S (12.81%); Found: C (62.4%), H (3.9%), N (11.0%), S (12.6%).

#### 3.1.7. Reactions of **8a**,**b** with Malononitrile (**12a**) and Ethyl cyanoacetate (**12b**): Formation of **13a**–**d**

To a solution of each of compounds **8a**,**b** (0.01 mol) in absolute ethanol (30 mL) containing a catalytic amount of piperidine was added either of malononitrile (**12a**; 0.66 g, 0.01 mol) or ethyl cyanoacetate (**12b**; 1.13 mL, 0.01 mol). The reaction mixture was heated under reflux for 3 h, under TLC monitoring, then cooled and poured onto ice-cold water. The solid product that separated was filtered off, dried and crystallized from ethanol.

*2-(4-(4-Chlorophenyl)-5,7-dioxo-6-phenyl-2-thioxo-1,2,3,4,6,7-hexahydropyrido[4,3-d]pyrimidin-8(5H)-ylidene)malononitrile* (**13a**): pale yellow crystals after crystallization from absolute ethanol; 52% yield; m.p. 223–225 °C; ^1^H-NMR: 3.20 (s, 1H, NH, D_2_O exchangeable), 4.80 (s, 1H, CH), 7.20–7.50 (m, 9H, Ar-H), 12.60 (s, 1H, NH, D_2_O exchangeable); ^13^C-NMR: 55.5 (pyrimidine C-4), 81.9 (methylidine C), 107.1 (CN), 112.1, 127.1, 127.9, 132.1, 132.9, 133.8, 134.7, 135.1, 137.3, 150.8, 154.3 (sp2 + aromatic C), 156.9, 160.7 (2CO), 178.0 (CS); IR ν: 3220, 3180 cm^−1^ (NH), 3060 (aromatic CH), 2850 (aliphatic CH), 2210 (CN), 1670, 1640 (2C=O), 1600, 1490 (aromatic C=C); MS (70 eV): M^+^ *m/z* 445 (12.2%) and 447 (4.7%). Anal. Calcd. for C_22_H_12_ClN_5_O_2_S (445.88): C (59.26%), H (2.71%), Cl (7.95%), N (15.71%), S (7.19%); Found: C (59.2%), H (2.5%), Cl (7.8%), N (15.6%), S (7.0%).

*2-(4-(4-Methoxyphenyl)-5,7-dioxo-6-phenyl-2-thioxo-1,2,3,4,6,7-hexahydropyrido[4,3-d]pyrimidin-8(5H)-ylidene)malononitrile* (**13b**): yellow crystals after crystallization from absolute ethanol; 50% yield; m.p. 214–216 °C; ^1^H-NMR: 3.10 (s, 1H, NH, D_2_O exchangeable), 3.80 (s, 1H, OCH_3_), 4.70 (s, 1H, CH), 6.90–7.50 (m, 9H, Ar-H), 12.60 (s, 1H, NH, D_2_O exchangeable); ^13^C-NMR: 54.7 (OCH_3_), 55.5 (pyrimidine C-4), 81.8 (methylidine C), 107.1 (CN), 112.1, 114.8, 127.3, 132.1, 132.9, 133.8, 134.6, 135.1, 137.3, 150.8, 154.3 (sp2 + aromatic C), 156.9, 160.7 (2CO), 178.1 (CS); IR ν: 3220, 3180 cm^−1^ (NH), 3060 (aromatic CH), 2850 (aliphatic CH), 2210 (CN), 1670, 1640 (2C=O), 1600, 1500 (aromatic C=C); MS (70 eV): M^+^ *m/z* 441 (15.1%); Anal. Calcd. for C_23_H_15_N_5_O_3_S (441.46): C (62.58%), H (3.42%), N (15.86%), S (7.26%); Found: C (62.6%), H (3.5%), N (15.7%), S (7.0%).

*Ethyl 2-(4-(4-chlorophenyl)-5,7-dioxo-6-phenyl-2-thioxo-1,2,3,4,6,7-hexahydropyrido[4,3-d]-pyrimidin-8(5H)-ylidene)-2-cyanoacetate* (**13c**): white crystals after crystallization from absolute ethanol; 42% yield; m.p. 188–190 °C; ^1^H-NMR: 1.20 (t, 3h, CH_3_), 3.00 (s, 1H, NH, D_2_O exchangeable), 4.30 (q, 2H, CH_2_), 5.10 (s, 1H, CH), 7.20–7.60 (m, 9H, Ar-H), 12.50 (s, 1H, NH, D_2_O exchangeable); ^13^C-NMR: 16.7 (CH_3_), 55.5 (pyrimidine C-4), 59.1 (CH_2_), 93.9 (methylidine C), 108.1 (CN), 123.4, 127.1, 127.9, 132.1, 132.9, 133.8, 134.6, 135.1, 137.3, 151.8, 157.1 (sp2 + aromatic C), 156.9, 160.7, 165.0 (3CO), 178.0 (CS); IR ν: 3220, 3180 cm^−1^ (NH), 3060 (aromatic CH), 2850 (aliphatic CH), 2230 (CN), 1710, 1670, 1640 (3C=O), 1600, 1490 (aromatic C=C); MS (70 eV): M^+^ *m/z* 492 (14.2%) and 494 (5.0%). Anal. Calcd. for C_24_H_17_ClN_4_O_4_S (492.93): C (58.48%), H (3.48%), Cl (7.19%), N (11.37%), S (6.50%); Found: C (58.3%), H (3.3%), Cl (7.1%), N (11.4%), S (6.4%).

*Ethyl 2-cyano-2-(4-(4-methoxyphenyl)-5,7-dioxo-6-phenyl-2-thioxo-1,2,3,4,6,7-hexahydropyrido-[4,3-d]pyrimidin-8(5H)-ylidene)acetate* (**13d**): white crystals after crystallization from absolute ethanol; 42% yield; m.p. 188–190 °C; ^1^H-NMR: 1.20 (t, 3h, CH_3_), 3.00 (s, 1H, NH, D_2_O exchangeable), 3.70 (s, 3H, CH_3_), 4.30 (q, 2H, CH_2_), 5.10 (s, 1H, CH), 6.90–7.60 (m, 9H, Ar-H), 12.30 (s, 1H, NH, D_2_O exchangeable); ^13^C-NMR: 16.7 (CH_3_), 53.1 (OCH_3_) 55.5 (pyrimidine C-4), 59.0 (CH_2_), 91.7 (methylidine C), 108.1 (CN), 117.7, 125.1, 126.9, 132.1, 132.9, 133.8, 133.6, 135.1, 137.3, 148.8, 156.6 (sp2 + aromatic C), 156.9, 160.7, 165.0 (3CO), 178.0 (CS); IR ν: 3220, 3180 cm^−1^ (NH), 3060 (aromatic CH), 2850 (aliphatic CH), 2230 (CN), 1710, 1670, 1640 (3C=O), 1600, 1490 (aromatic C=C); MS (70 eV): M^+^ *m/z* 488 (13.9%); Anal. Calcd. for C_25_H_20_N_4_O_5_S (488.52): C (61.47%), H (4.13%), N (11.47%), S (6.56%); Found: C (61.5%), H (4.0%), N (11.4%), S (6.4%).

#### 3.1.8. Reaction of **13a**–**d** with Hydrazine Hydrate and Thiourea: Formation of **14a**–**d** and **15a**–**d**

**Method A**: To a solution of each of compounds **13a**–**d** (0.01 mol) in absolute ethanol (30 mL) containing a catalytic amount of piperidine was added hydrazine (0.32 mL, 0.01 mol) or thiourea (0.76 g, 0.01 mol). The reaction mixture was heated under reflux for 3 h, under TLC monitoring, then cooled and poured onto ice-cold water. The solid product that separated was filtered off, dried and crystallized from ethanol.

**Method B**: The same reactants of method A were heated in microwave oven at 500 W and 140 °C for 7 min. The reaction mixture was treated similar to method A to obtain compounds **14a**–**d** and **15a**–**d**.

*5-Amino-4'-(4-chlorophenyl)-5',7'-dioxo-6'-phenyl-2'-thioxo-2,2',3',4,4',5',6',7'-octahydro-1'H-spiro[pyrazole-3,8'-pyrido[4,3-d]pyrimidine]-4-carbonitrile* (**14a**): white crystals after crystallization from absolute dioxane; 37% yield (Method A) and 81% (Method B); m.p. 235–237 °C; ^1^H-NMR: 3.20 (s, 1H, NH, D_2_O exchangeable), 4.10 (s, 1H, pyrazole H-4), 5.10 (s, 1H, pyrimidine H-4), 6.2 (s, 1H, NH, D_2_O exchangeable), 7.20–7.50 (m, 9H, Ar-H), 9.1 (s, 2H, NH_2_, D_2_O exchangeable), 12.60 (s, 1H, NH, D_2_O exchangeable); ^13^C-NMR: 39.5 (pyrazole C-4), 55.4 (pyrimidine C-4), 58.9 (spiro-C), 107.1 (CN), 110.3, 116.1, 126.9, 131.8, 132.7, 133.8, 134.6, 135.1, 137.3, 143.1, 154.3 (sp2 + aromatic C), 156.9, 160.7 (2CO), 178.0 (CS); IR ν: 3350, 3220, 3180 cm^−1^ (broad, NH), 3060 (aromatic CH), 2850 (aliphatic CH), 2200 (CN), 1670, 1640 (2C=O), 1600, 1490 (aromatic C=C);MS (70 eV): M^+^ *m/z* 477 (9.2%) and 447 (3.5%); Anal. Calcd. for C_22_H_16_ClN_7_O_2_S (477.93): C (55.29%), H (3.37%), Cl (7.42%), N (20.52%), S (6.71%); Found: C (55.1%), H (3.1%), Cl (7.3%), N (20.4%), S (6.6%).

*5-Amino-4'-(4-methoxyphenyl)-5',7'-dioxo-6'-phenyl-2'-thioxo-2,2',3',4,4',5',6',7'-octahydro-1'H-spiro[pyrazole-3,8'-pyrido[4,3-d]pyrimidine]-4-carbonitrile* (**14b**): white crystals after crystallization from dil. dioxane; 42% yield (Method A) and 84% (Method B); m.p. 229–231 °C; ^1^H-NMR: 3.00 (s, 1H, NH, D_2_O exchangeable), 3.70 (s, 3H, OCH_3_), 4.10 (s, 1H, pyrazole H-4), 5.10 (s, 1H, pyrimidine H-4), 6.2 (s, 1H, NH, D_2_O exchangeable), 7.20–7.50 (m, 9H, Ar-H), 9.4 (s, 2H, NH_2_, D_2_O exchangeable), 12.50 (s, 1H, NH, D_2_O exchangeable); ^13^C-NMR: 39.5 (pyrazole C-4), 53.7 (OCH_3_), 55.4 (pyrimidine C-4), 58.9 (spiro-C), 107.1 (CN), 112.7, 114.3, 126.9, 131.8, 132.7, 133.8, 134.6, 135.1, 137.3, 143.1, 154.3 (sp2 + aromatic C), 156.9, 160.7 (2CO), 178.0 (CS); IR ν: 3350, 3220, 3180 cm^−1^ (broad, NH), 3060 (aromatic CH), 2850 (aliphatic CH), 2200 (CN), 1670, 1640 (2C=O), 1600, 1490 (aromatic C=C); MS (70 eV): M^+^ *m/z* 473 (10.8%); Anal. Calcd. for C_23_H_19_N_7_O_3_S (477.93): C (58.43%), H, 4.04; N, 20.71; S, 6.77; Found: C (58.3%), H (4.1%), N (20.5%), S (6.6%).

*4'-(4-Chlorophenyl)-5-hydroxy-5',7'-dioxo-6'-phenyl-2'-thioxo-2,2',3',4,4',5',6',7'-octahydro-1'H-spiro-[pyrazole-3,8'-pyrido[4,3-d]pyrimidine]-4-carbonitrile* (**14c**): white crystals after crystallization from absolute ethanol; 45% yield (Method A) and 89% (Method B); m.p. 258–260 °C; ^1^H-NMR: 3.40 (s, 1H, NH, D_2_O exchangeable), 4.50 (s, 1H, pyrazole H-4), 5.40 (s, 1H, pyrimidine H-4), 6.2 (s, 1H, NH, D_2_O exchangeable), 7.20–7.50 (m, 9H, Ar-H), 11.1 (s, 1H, OH, D_2_O exchangeable), 12.60 (s, 1H, NH, D_2_O exchangeable); ^13^C-NMR: 41.1 (pyrazole C-4), 55.4 (pyrimidine C-4), 58.9 (spiro-C), 108.9 (CN), 110.3, 115.1, 126.9, 128.8, 130.1, 132.5, 134.6, 135.1, 137.3, 143.1, 154.3 (sp2 + aromatic C), 156.9, 160.7 (2CO), 178.0 (CS); IR ν: 3400, 3270, 3180 cm^−1^ (broad, NH + OH), 3060 (aromatic CH), 2850 (aliphatic CH), 2200 (CN), 1670, 1640 (2C=O), 1600, 1490 (aromatic C=C); MS (70 eV): M^+^ *m/z* 478 (10.7%) and 480 (3.8%); Anal. Calcd. for C_22_H_15_ClN_6_O_3_S (478.91): C (55.17%), H (3.16%), Cl (7.40%), N (17.55%), S (6.70%); Found: C (55.0%), H (3.2%), Cl (7.3%), N (17.3%), S (6.6%).

*5-Hydroxy-4'-(4-methoxyphenyl)-5',7'-dioxo-6'-phenyl-2'-thioxo-2,2',3',4,4',5',6',7'-octahydro-1'H-spiro[pyrazole-3,8'-pyrido[4,3-d]pyrimidine]-4-carbonitrile* (**14d**): white crystals after crystallization from absolute ethanol; 44% yield (Method A) and 82% (Method B); m.p. 237–239 °C; ^1^H-NMR: 3.30 (s, 1H, NH, D_2_O exchangeable), 3.80 (s, 3H, OCH_3_), 4.10 (s, 1H, pyrazole H-4), 5.10 (s, 1H, pyrimidine H-4), 6.2 (s, 1H, NH, D_2_O exchangeable), 7.20–7.50 (m, 9H, Ar-H), 10.8 (s, 1H, OH, D_2_O exchangeable, 12.50 (s, 1H, NH, D_2_O exchangeable); ^13^C-NMR: 39.5 (pyrazole C-4), 53.8 (OCH_3_), 55.4 (pyrimidine C-4), 58.9 (spiro-C), 108.9 (CN), 112.7, 124.8, 126.9, 128.8, 132.1, 133.3, 134.6, 135.1, 137.3, 144.6, 154.3 (sp2 + aromatic C), 156.9, 160.7 (2CO), 178.0 (CS); IR ν: 3350, 3200, 3150 cm^−1^ (broad, NH + OH), 3060 (aromatic CH), 2850 (aliphatic CH), 2200 (CN), 1670, 1640 (2C=O), 1600, 1490 (aromatic C=C); MS (70 eV): M^+^ *m/z* 474 (12.0%); Anal. Calcd. for C_23_H_18_N_6_O_4_S (474.49): C (58.22%), H (3.82%), N (17.71%), S (6.76%); Found: C (58.1%), H (3.6%), N (17.5%), S (6.7%).

*6'-Amino-4-(4-chlorophenyl)-5,7-dioxo-6-phenyl-2,2'-dithioxo-2,3,3',4,5,5',6,7-octahydro-1H,2'H-spiro[pyrido[4,3-d]pyrimidine-8,4'-pyrimidine]-5'-carbonitrile* (**15a**): white crystals after crystallization from dil. DMF; 47% yield (Method A) and 88% (Method B); m.p. 240–242 °C; ^1^H-NMR: 3.00 (s, 1H, NH, D_2_O exchangeable), 3.40 (s, 1H, spiro-pyrimidine H-4), 5.10 (s, 1H, pyrimidine H-4), 5.50 (s, 1H, NH, D_2_O exchangeable), 7.20–7.50 (m, 9H, Ar-H), 8.70 (s, 2H, NH_2_, D_2_O exchangeable), 12.00 (s, 1H, NH, D_2_O exchangeable); ^13^C-NMR: 29.0 (spiro-pyrimidine C-5), 55.4 (pyrimidine C-4), 63.9 (spiro-C), 107.8 (CN), 114.3, 119.5, 126.9, 128.8, 132.7, 133.8, 134.6, 136.1, 138.1, 143.1, 154.3 (sp2 + aromatic C), 156.9, 160.7 (2CO), 178.0, 181.0 (2CS); IR ν: 3350, 3220, 3180 cm^−1^ (broad, NH), 3060 (aromatic CH), 2850 (aliphatic CH), 2200 (CN), 1670, 1640 (2C=O), 1600, 1490 (aromatic C=C);MS (70 eV): M^+^ *m/z* 522 (10.0%) and 524 (3.8%); Anal. Calcd. for C_23_H_16_ClN_7_O_2_S_2_ (522.00): C (52.92%), H (3.09%), Cl (6.79%), N (18.78%), S (12.29%); Found: C (52.8%), H (3.2%), Cl (6.8%), N (18.9%), S (12.1%).

*6'-Amino-4-(4-methoxyphenyl)-5,7-dioxo-6-phenyl-2,2'-dithioxo-2,3,3',4,5,5',6,7-octahydro-1H,2'H-spiro[pyrido[4,3-d]pyrimidine-8,4'-pyrimidine]-5'-carbonitrile* (**15b**): white crystals after crystallization from dil. DMF; 43% yield (Method A) and 87% (Method B); m.p. 226–228 °C; ^1^H-NMR: 2.80 (s, 1H, NH, D_2_O exchangeable), 3.50 (s, 1H, spiro-pyrimidine H-4), 3.80 (s, 1H, OCH_3_), 5.00 (s, 1H, pyrimidine H-4), 5.50 (s, 1H, NH, D_2_O exchangeable), 6.80–7.50 (m, 9H, Ar-H), 8.50 (s, 2H, NH_2_, D_2_O exchangeable), 12.20 (s, 1H, NH, D_2_O exchangeable); ^13^C-NMR: 27.8 (spiro-pyrimidine C-5), 52.6 (OCH_3_), 55.7 (pyrimidine C-4), 64.2 (spiro-C), 107.8 (CN), 112.5, 116.2, 125.2, 127.0, 128.4, 129.8, 131.6, 136.1, 138.1, 139.0, 154.2 (sp2 + aromatic C), 156.9, 160.7 (2CO), 178.0, 181.0 (2CS); IR ν: 3350, 3200, 3160 cm^−1^ (broad, NH), 3080 (aromatic CH), 2850 (aliphatic CH), 2200 (CN), 1670, 1640 (2C=O), 1600, 1500 (aromatic C=C); MS (70 eV): M^+^ *m/z* 517 (11.3%); Anal. Calcd. for C_24_H_19_N_7_O_3_S_2_ (517.58): C (55.69%), H (3.70%), N (18.94%), S (12.39%); Found: C (55.6%), H (3.5%), N (18.9%), S (12.1%).

*4-(4-Chlorophenyl)-6'-hydroxy-5,7-dioxo-6-phenyl-2,2'-dithioxo-2,3,3',4,5,5',6,7-octahydro-1H,2'H-spiro[pyrido[4,3-d]pyrimidine-8,4'-pyrimidine]-5'-carbonitrile* (**15c**): white crystals after crystallization from dioxane; 51% yield (Method A) and 90% (Method B); m.p. 255–257 °C; ^1^H-NMR: 3.20 (s, 1H, NH, D_2_O exchangeable), 3.50 (s, 1H, spiro-pyrimidine H-4), 5.10 (s, 1H, pyrimidine H-4), 5.80 (s, 1H, NH, D_2_O exchangeable), 7.20–7.50 (m, 9H, Ar-H), 11.10 (s, 1H, OH, D_2_O exchangeable), 12.40 (s, 1H, NH, D_2_O exchangeable); ^13^C-NMR: 29.0 (spiro-pyrimidine C-5), 55.4 (pyrimidine C-4), 63.9 (spiro-C), 107.8 (CN), 113.9, 119.8, 127.2, 128.8, 132.4, 133.8, 134.5, 136.5, 138.6, 152.1, 155.3 (sp2 + aromatic C), 158.9, 162.7 (2CO), 178.0, 180.5 (2CS); IR ν: 3350, 3220, 3180 cm^−1^ (broad, NH), 3050 (aromatic CH), 2850 (aliphatic CH), 2200 (CN), 1670, 1640 (2C=O), 1600, 1490 (aromatic C=C); MS (70 eV): M^+^ *m/z* 522 (14.0%) and 524 (4.7%); Anal. Calcd. for C_23_H_15_ClN_6_O_3_S_2_ (522.99): C (52.82%), H (2.89%), Cl (6.78%), N (16.07%), S (12.26%); Found: C (52.6%), H (2.9%), Cl (6.6%), N (15.9%), S (12.1%).

*6'-Hydroxy-4-(4-methoxyphenyl)-5,7-dioxo-6-phenyl-2,2'-dithioxo-2,3,3',4,5,5',6,7-octahydro-1H,2'H-spiro[pyrido[4,3-d]pyrimidine-8,4'-pyrimidine]-5'-carbonitrile* (**15d**): white crystals after crystallization from dioxane; 47% yield (Method A) and 91% (Method B); m.p. 238–240 °C; ^1^H-NMR: 2.90 (s, 1H, NH, D_2_O exchangeable), 3.60 (s, 1H, spiro-pyrimidine H-4), 3.90 (s, 1H, OCH_3_), 5.00 (s, 1H, pyrimidine H-4), 5.50 (s, 1H, NH, D_2_O exchangeable), 6.80–7.50 (m, 9H, Ar-H), 10.80 (s, 1H, OH, D_2_O exchangeable), 12.10 (s, 1H, NH, D_2_O exchangeable); ^13^C-NMR: 27.2 (spiro-pyrimidine C-5), 54.4 (OCH_3_), 57.1 (pyrimidine C-4), 64.0 (spiro-C), 108.4 (CN), 116.5, 119.6, 125.0, 127.4, 128.8, 130.3, 132.6, 136.1, 138.1, 139.0, 153.1 (sp2 + aromatic C), 158.9, 160.7 (2CO), 178.0, 181.3 (2CS); IR ν: 3350, 3200, 3160 cm^−1^ (broad, NH), 3080 (aromatic CH), 2850 (aliphatic CH), 2200 (CN), 1670, 1640 (2C=O), 1600, 1500 (aromatic C=C);MS (70 eV): M^+^ *m/z* 518 (14.0%), 516 (5.1%); Anal. Calcd. for C_24_H_18_N_6_O_4_S_2_ (518.57): C (55.59%), H (3.50%), N (16.21%), S (12.37%); Found: C (55.4%), H (3.4%), N (16.3%), S (12.3%).

### 3.2. Antimicrobial Screening

The newly synthesized heterocyclic compounds were tested for their antimicrobial activity against the following microorganisms: (a) Gram-negative: *Escherichia coli* and *Pseudomonas putide*; (b) Gram-positive: *Bacillus subtilis* and *Streptococcus lactis*; (c) Fungi: *Aspergillus niger* and *Penicillium* sp.; (d) Yeast: *Candida albicans. Media:* Three types of specific media were used in this study:

*Medium 1*: Nutrient Medium for bacteria, consisting of (g/L distilled water): peptone, 5 and meat extract, 3. pH was adjusted to 7.0.
*Medium 2*: Potato Dextrose Medium for fungi, consisting of (g/L distilled water): Infusion from potatoes, 4 and D(+)glucose, 20. pH was adjusted to 5.5.
*Medium 3*: Universal Medium for yeast, consisting of (g/L distilled water): yeast extract, 3; malt extract, 3; peptone, 5 and glucose, 10. pH was adjusted to 5.5.

For solid media, 2% agar was added. All media were sterilized at 121 °C for 20 min.

### 3.3. Procedure (Filter Paper Diffusion Method) [27]

Proper concentrations of microbial suspensions were prepared from 1 (for bacteria) to 3 (for yeast and fungi)-day-old liquid stock cultures incubated on a rotary shaker (100 rpm). In the case of fungi, five sterile glass beads were added to each culture flask. The mycelia were then subdivided by mechanical stirring at speed No. 1 for 30 min. Turbidity of microorganisms was adjusted with a spectrophotometer at 350 nm to give an optical density of 1.0. Appropriate agar plates were aseptically surface inoculated uniformly by a standard volume (*ca.* 1 mL) of the microbial broth culture of the tested microorganism, namely *E. coli*, *P. putida*, *B. subtilis*, *S. lactis*, *A. niger*, *Penicillium* sp. and *C. albicans*. Whatman No. 3 filter paper discs of 10 mm diameter were sterilized by autoclaving for 15 min at 121 °C. Test compounds were dissolved in 80% ethyl alcohol to give final concentration of 5 μg/mL. The sterile discs were impregnated with the test compounds (5 μg/disc). After the impregnated discs have been air dried, they were placed on the agar surface previously seeded with the organism to be tested. Discs were gently pressed with forceps to insure thorough contact with the media. Three discs were arranged per dish, suitably spaced apart, *i.e.*, the discs should be separated by a distance that is equal to or slightly greater than the sum of the diameters of inhibition produced by each disc alone. Each test compound was conducted in triplicate. Plates were kept in the refrigerator at 5 °C for 1 h to permit good diffusion before transferring them to an incubator at 37 °C for 24 h for bacteria and at 30 °C for 72 h for yeast and fungi.
